# Effects of limiting environmental conditions on functional traits of *Hedera helix* L. vegetative shoots

**DOI:** 10.3389/fpls.2024.1464006

**Published:** 2024-11-07

**Authors:** Olena Blinkova, Katarzyna Rawlik, Andrzej M. Jagodziński

**Affiliations:** ^1^ Institute of Dendrology, Polish Academy of Sciences, Kórnik, Poland; ^2^ Educational and Research Institute of Natural and Agrarian Sciences, Taras Shevchenko Lugansk National University, Poltava, Ukraine

**Keywords:** ivy, vegetative individuals, soil moisture, water content, specific leaf area, light availability

## Abstract

*Hedera helix* L. is a widespread liana that significantly influences forest ecosystems in temperate zones, exhibiting high adaptability to varying soil moisture and light levels. In this study, it was confirmed that *H. helix* dominates the herbaceous layer of the Kórnik Arboretum (Poland), with clear links between its above-ground biomass and key environmental factors. The study revealed that, under intense soil shading, the leaf to stem biomass ratio was disproportional, favoring leaves. Leaf and stem water content reflected the plant’s adaptation to soil moisture, aligning with its field capacity. Strong relationships were found between leaf water content and soil moisture, while the correlations between leaf water content and light availability were weaker. The study also confirmed positive relationships between daily light integral and leaf water content, with a less pronounced effect on stem water content. These results enhance understanding of *H. helix*’s role in temperate forests and its impact on ecosystem regeneration.

## Introduction

Plants residing in tree canopies are found in forest ecosystems across all climate zones (Hargis et al., 2019). As lianas influence many ecological processes within forest ecosystems (Collins et al., 2016; Reichstein et al., 2014), studying these plants is important. Similar studies provide further insight into the development and growth of woody vines but also improves understanding of relationships and patterns of the functioning and the integrity of the forest ecosystem (Schnitzer and Bongers, 2011).

Most of the previous studies on woody vines specialized in ecological investigation of perennial plants with woody stems in tropical and subtropical zones (Schnitzer and Bongers, 2011; Schnitzer et al., 2012; van der Heijden et al., 2022). The spread of lianas can alter the composition (Tobin et al., 2012), biological variability (Alvarez-Cansino et al., 2015), net primary production (Kaneko and Homma, 2006), and structure of forest communities (Schnitzer et al., 2012). Although there are studies on the distribution and ecological characteristics of woody liana species in the neotropical region, detailed and systematic data from the palearctic region (temperate biomes) remain relatively limited, particularly regarding biomass dynamics and environmental interactions. The percentage share of liana communities in temperate woodlands is smaller than in tropical forests, reaching 10% (Schnitzer and Bongers, 2011; Schnitzer et al., 2012). However, an increase in the number of liana individuals was recorded in temperate forests (Wyka et al., 2023). These changes are associated with a warming climate, the wide ecological niches of invasive species, and disturbances to the integrity of forest habitats (Yuan et al., 2009; Schnitzer et al., 2012). Specifically, factors like canopy openness, soil moisture, and average canopy height have an impact on number of individuals and their distribution (Yuan et al., 2009). Liana spread was greatest near the edges of woodlands, and was largely dependent on habitat modification and significantly negatively associated with vegetation biomass (Laurance et al., 2001). Photosynthetic capacity of lianas can serve as an indicator for forest and park productive parameters (Bi et al., 2004). Liana abundances increase in response to human-induced forest disturbance (Putz, 1983). Although an increase in the abundance of lianas was detected in the understories of deciduous temperate forests in Europe (Perring et al., 2020), detailed studies on liana biomass are still lacking. Previous research on forest biomass has emphasized the importance of direct *in situ* measurements for assessing above-ground biomass rather than relying solely on allometric equations (Asner et al., 2013; Smart et al., 2017). However, debate continues over which functional traits are most closely associated with above-ground biomass (Putz, 1983; Wyka et al., 2013; Zotz et al., 2006).

*Hedera helix* L. is one of the most widespread woody vines across Europe (Castagneri et al., 2013). *H. helix* belongs to the *Araliaceae* family (Ackerfield and Wen, 2003; Metcalfe, 2005). The species is especially abundant in its vegetative (juvenile) form (Bunk et al., 2019; Metcalfe, 2005; Okerman, 2000). During this phase, *H. helix* reproduces clonally and does not produce seeds, making vegetative propagation crucial for its successful spread (Okerman, 2000; Schnitzer et al., 2012). It thrives under a wide range of edaphic conditions (Ellenberg, 1998; Ellenberg and Leuschner, 2010), favoring moist, fertile soils but also tolerating environments ranging from relatively dry to moderately damp. *H. helix* is most frequently found in clay-rich soils and is less common in nutrient-poor, well-drained sandy soils (Thomas, 1998; Metcalfe, 2005). The primary environmental factors limiting its abundance are soil moisture and light availability (Ellenberg, 1998; Ellenberg and Leuschner, 2010; Hoflacher and Bauer, 2006; Koziarz, 2015; Sack and Grubb, 2002; Schnitzler and Heuze, 2006).

An increase of *H. helix* distribution in Central Europe was detected in different natural and artificial habitats within the last several years. On average, the occurrence of ivy has grown by 14% across 40 European study sites (Perring et al., 2020). In the flora of Europe, ivy (a generalist liana) stands out as an evergreen species and a potentially important component of forest ecosystem composition and productivity (Metcalfe, 2005). The old-growth forests in Central Europe are becoming fragmented as areas covered by second-growth forests increase. Consequently, the impact of widespread *H. helix* lianas in the remaining primary forest fragments and in forest regrowth is expected to increase in the future. Increasing distribution of ivy could relate to diaspore impact. Transition to the adult form of *H. helix* only occurs once the liana has reached a certain height or develops in light, and has therefore moved out of the understory layer (Perring et al., 2020).

The eastern region of Poland represents the easternmost limit of *H. helix* distribution within Europe (Koziarz, 2015). *H. helix* is typically found on the edges of deciduous and mixed forests, thriving in fresh and moist habitats, particularly in oak-hornbeam and riparian alder-ash forests throughout the country (Koziarz, 2015). Due to its frequent vegetative reproduction, *H. helix* has become an increasingly expansive species, with a growing number of habitats now supporting flowering individuals (Kucharski et al., 2019). Ivy often forms dense thickets in the lower story of forests. It may inhibit the growth of co-occurring native vegetation (Zotz et al., 2006).

The consistent Europe-wide pattern of ivy spread also suggests that large-scale drivers may influence its distribution (Senf et al., 2018). The evergreen nature of *H. helix* can extend the growing season of the vegetation beneath it, affecting tree regeneration and altering forest canopy composition (Ladwig and Meiners, 2009). Ivy can promote biological diversity in landscapes through the provision of useful habitat and resources for other organisms (Metcalfe, 2005). Studies have shown that lianas, including ivy, may drive above-ground biomass productivity in the undergrowth shrub layer (Wasof et al., 2018). The vegetative form of lianas contribute a large proportion of the community of arboreal understory plants in old-growth forests (Roeder and Meyer, 2022). While previous research has explored the environmental impacts of *H. helix*, no studies have specifically examined how its above-ground biomass and water content respond to varying light and soil moisture conditions in temperate forests. Therefore, understanding the relationship between limiting environmental factors and juvenile above-ground biomass of ivy is essential for explaining its colonization success and wide ecological niche.

Considering the conclusions of preceding studies, we hypothesized that: 1) above-ground biomass of *H. helix* vegetative shoots is positively associated with key limiting environmental factors such as soil moisture and light; 2) *H. helix* adjusts its above-ground biomass allocation, particularly the leaf-to-stem ratio, in response to varying light conditions, favoring leaf production in shaded environments; and 3) water content in the leaves and stems of *H. helix* correlates with soil moisture and light availability, helping the plant maintain water balance and survive in fluctuating environmental conditions.

This study examines the functional traits of *H. helix* vegetative shoots in different subpopulations within a temperate forest, focusing on how they respond to variations in key limiting ecological factors, specifically soil moisture and light availability.

## Methods

### Study design

The study was conducted from July to August 2022 at the Kórnik Arboretum (Poland; 52.2448°N, 17.09698°E, 75 m a.s.l.; Appx.) located in Central Poland. Fieldwork took place from 10:00 to 12:00 AM. Eleven 50×50 m experimental plots (EP) were laid out in different environmental conditions and dominant soil types within the study area (Appx.). Species identification was performed in the field and later verified in a laboratory. The analysis was carried out at the level of loci of ivy subpopulations (Appx.). Within each plot, six randomly selected subplots (5×5 m) were established for phytoindicative data collection.

The Kórnik Arboretum represents a variety of microhabitats with differing moisture and light conditions, ranging from shaded forest understories to more open areas. *H. helix* thrives in these diverse habitats, making it essential to select bioindicators that accurately reflect these varying environmental gradients. By integrating indirect phytoindicative values with direct measurements, we ensured the representativeness of data across all experimental plots. These methods align with established protocols in ecological field research, enabling comparison with other studies.

### Phytoindicative study

The bioindication method was used to determine environmental conditions (Ellenberg, 1998; Didukh, 2012). This method is applicable for identification of edaphic factors based on the plant species composition. The environmental indicators of soil properties were determined using standardized scales of environmental amplitudes (Ellenberg, 1998; Ellenberg and Leuschner, 2010; Holtland et al., 2010). Ellenberg-type indicator values rank plant species according to their ecological niches (Tichy et al., 2023). These indicator values for abiotic environmental factors are continuously updated and refined. Phytoindicative values (Ellenberg and Leuschner, 2010) are commonly used for rapid estimation of the environmental characteristics (Holtland et al., 2010). Studies suggested that correlations exist between community means (weighted and averaged) measured by phytoindicative values and locally calculated environmental variables (Ellenberg, 1998; Diekmann, 2003). The indicator values are available at https://sci.muni.cz/botany/juice. The phytoindicative value of soil moisture (HD) and variability of soil moisture (FH) were strongly correlated with real moisture reserves in the soil (Halarewicz et al., 2021), while soil light (LC) was an important factor affecting the community composition (Ellenberg, 1998; Ellenberg and Leuschner, 2010). Therefore, the effects of these edaphic factors were analyzed for this study. Soil moisture (HD) ranges from 1 for dry soils to 23 for wet soils. Variability of soil moisture (FH) is an essential element in the dispersion of species in relation to the fluctuation of moisture conditions. Variability of soil moisture takes values from 1 (species that grow in constant conditions of the same humidity) to 9 (species that grow in conditions of constantly changing humidity). Soil light (LC) takes values from 1 on heavily shaded soils to 9 on soils receiving full sunlight.

The means of the indicator values were calculated as arithmetic averages based on the presence or absence of species on EPs (aHD, aFH, aLC) as well as weighted means based on plant cover (wHD, wFH, wLC).

### Light intensity study

Photosynthetically active radiation (PAR) was measured using a Quantitherm PAR device (Hansatech Instruments). The light intensity parameter was measured at a height of 10 cm above creeping ivy individuals on the subplots. The Daily Light Integral (DLI) indicates the total amount of PAR received (mol×m^-2^×d^-1^).

### Biomass measurements

A representative subset of vegetative liana individuals was sampled on the study area following the methods outlined by Condit (2008). Above-ground biomass included both leaves and stems. We used the destructive method for estimating plant biomass (*H. helix* and forbs) on 1 m^2^ plots (four-fold replication on EP). Above-ground plant organs were separated using pruning shears, tagged in the laboratory, and weighed to measure water loss. The collected plant material (leaves, stems) of *H. helix* individuals was oven-dried at 65°C in the ovens (ULE 600 and UF450, Memmert GmbH + Co. KG, Germany). All dry biomass samples of *H. helix* were weighed. Additionally, we recorded the above-ground fresh and dry biomass of herbaceous species without separating them into parts, as well as the fresh and dry biomass of shoot litter.

### Productivity traits

Leaf Water Content (LWC), Stem Water Content (STWC), and Above-ground Biomass Water Content (ABWC) were calculated as follows (Garnier and Laurent, 1994; Lin et al., 2019):


(1) $$LWC=\frac{LWF-LDM}{LDW}\;\times 100\%,$$

(2) $$STWC=\frac{STFW-STDM}{STFW}\;\times 100\%,$$

(3) $$ABWC=\frac{(LWF-LDM)+(STFW-STDM)}{LFW+STFW}\;\times 100\%,$$

where: LWF is the leaf fresh mass (g); LDM is the leaf dry mass (g); STFW is the stem fresh mass (g); and STDM is the stem dry mass (g).

Specific Leaf Area (SLA) is a salient ecological trait as it is associated with plant development processes. SLA quickly responds to environmental changes. SLA was determined as:


(4) $$SLA=\frac{LA}{W},$$

where: LA is the leaf area (cm^2^); W is the leaf dry mass (g).

Leaf Area was determined by a non-destructive method, based on morphometric measurements of leaf traits following the method of Rosu and Sala (2022):


(5) $$LA=L_{l}\times W_{l}\times CF,$$

where: L_l_ is the leaf length (cm); W_l_ is the leaf width (cm); CF is the specific correction factor for *H. helix* leaves. The optimal value of the correction factor (CF) specific to *H. helix* leaves was calculated according to the model proposed by Sala et al. (2015).

### Statistical analysis

Descriptive statistical analyses of the data were used for the analytical overview of the obtained fresh/dry above-ground biomass (leaf/stem) of ivy, fresh/dry above-ground biomass of forbs, fresh/dry shoot litter biomass and bioindicative values (min, max, average, standard deviation, standard error); variability (coefficient of variation) was also quantified. To assess the relationships between the water content of various plant parts (LWC, STWC, ABWC), phytoindicative values of soil moisture conditions (HD, FH) and soil light (LC), we performed correlation analysis (Pearson’s method; p<0.05, p<0.01, p<0.001). Linear regression analysis was used to predict the value of fresh/dry leaf/stem biomass of *H. helix* per area on the value of DLI/SLA as well as other interdependence links between variables. We used a generalized scatter plot matrix to estimate the links between functional traits: DLI, LWC, STWC and ABWC. For the analytical processing of the field and laboratory data, the calculation was performed using OriginPro 2022 software.

## Results

In the recent studies (Blinkova et al., 2023), only individuals of *H. helix* in the vegetative phase of growth were recorded in the study area. No generative individuals of *H. helix* with flowers, fruits and seeds were found. The studied clusters of ivy subpopulations were incomplete, as generative individuals of the species were not taken into account. *H. helix* was a habitat-forming species of the shrub layer and forest floor plant communities in the Arboretum. The individuals of *H. helix* were dominant in the artificial plant community (by projected foliage cover/above-ground biomass), regardless of the age and developmental stage of individuals. The horizontal composition of the studied subpopulations appeared to be random and rare.

### Phytoindicative analysis of limiting environmental conditions

Phytoindicative evaluation of soil moisture conditions revealed rather narrow ranges in values. Mesophytic conditions were predominant across the EPs, as assessed by both weighted and arithmetic HD and FH (**Table 1**). The weighted mean index values for soil moisture (wHD) ranged from 11.2 to 11.7, while the arithmetic mean index values (aHD) ranged from 13.0 to 13.4. Available soil water for plants at the level of 100–l45 mm was recorded, consistent with the indicative weighted and averaged environmental indicators. The weighted mean index values for variability of soil moisture (wFH) ranged from 4.9 to 5.3, and the arithmetic mean index values (aFH) ranged from 5.0 to 5.6. The wFH/aFH ratio indicated favorable abiotic conditions for plants in fresh forest-meadow habitats, characterized by moderately uneven moisture in the O-A horizons of the soil, which is maintained by atmospheric precipitation and melt water. In contrast to moisture conditions, the values for soil light (LC) showed more variation (**Table 2**). The weighted mean index values for soil light ranged from 4.1 to 6.2, while the arithmetic mean index values ranged from 4.2 to 6.3.

**Table 1:** Phytoindicative analysis of soil moisture and variability of soil moisture.

№ EP	wHD*				aHD*				wFH*				aFH*			
	X_mean_	X_min_	X_max_	σ	X_mean_	X_min_	X_max_	σ	X_mean_	X_min_	X_max_	σ	X_mean_	X_min_	X_max_	σ
1	11.7	11.1	12.1	0.229	13.2	12.8	13.8	0.445	5.1	4.8	5.7	0.213	5.6	5.2	6.0	0.223
2	11.6	11.4	12.1	0.204	13.4	12.6	13.8	0.476	5.2	5.0	5.5	0.335	5.4	5.2	5.8	0.249
3	11.7	11.5	12.5	0.471	13.4	12.5	13.7	0.419	4.9	4.5	5.1	0.234	5.4	4.8	5.8	0.360
4	11.5	11.3	12.0	0.177	13.0	12.4	13.4	0.371	5.3	5.0	5.5	0.151	5.2	5.0	5.7	0.221
5	11.6	11.5	12.0	0.151	13.4	12.8	13.6	0.323	5.3	4.5	5.7	0.243	5.1	4.8	5.5	0.214
6	11.4	11.0	12.2	0.217	13.0	12.4	13.3	0.298	5.3	5.0	5.6	0.197	5.1	4.6	5.4	0.279
7	11.8	11.5	12.5	0.289	13.4	12.6	13.4	0.312	5.3	4.9	5.5	0.216	5.0	4.5	5.8	0.386
8	11.5	11.1	12.5	0.397	13.1	12.5	13.4	0.235	5.2	5.0	5.4	0.119	5.3	4.9	5.8	0.219
9	11.5	11.0	12.5	0.410	13.1	12.6	13.4	0.315	5.3	4.8	5.5	0.172	5.2	4.9	5.8	0.247
10	11.2	11.0	11.8	0.327	13.0	12.6	13.6	0.402	5.5	5.2	5.7	0.206	5.6	5.0	5.8	0.119
11	11.4	11.0	12.0	0.299	13.2	12.4	13.6	0.398	5.3	5.1	5.6	0.160	5.4	4.8	5.8	0.276

*: a – arithmetic, w – weighted; HD – soil moisture, FH – variability of soil moisture.

**Table 2:** Phytoindicative analysis of soil light.

№ EP	wLC*				aLC*			
	X_mean_	X_min_	X_max_	σ	X_mean_	X_min_	X_max_	σ
1	5.0	4.8	5.3	0.236	5.2	4.6	5.6	0.355
2	5.9	5.4	6.2	0.616	5.8	5.0	6.2	0.516
3	4.1	3.5	4.5	0.272	4.2	3.5	4.5	0.412
4	6.2	5.5	6.8	0.621	6.3	5.5	6.8	0.693
5	4.2	3.7	4.4	0.204	4.4	3.5	4.6	0.276
6	5.3	4.8	5.7	0.301	5.0	4.6	5.8	0.342
7	5.1	4.8	5.6	0.324	5.0	4.6	5.8	0.442
8	5.2	4.9	5.4	0.147	5.4	5.0	5.6	0.153
9	5.1	4.6	5.4	0.233	5.0	4.5	5.8	0.276
10	5.8	5.2	6.2	0.262	6.1	5.1	6.3	0.432
11	5.3	4.9	5.7	0.145	5.4	5.0	5.8	0.176

*: a – arithmetic, w – weighted; LC – soil light.

### Above-ground biomass of *H. helix* vegetative shoots

The main functional traits of the above-ground biomass of ivy during its vegetative phase of growth on the EPs are presented in **Table 3**. The fresh biomass of *H. helix* vegetative shoots accounted for 90% of the total above-ground biomass on the studied EPs. The maximum leaf biomass recorded was 594.52 g×m^-2^, while the minimum was 145.14 g×m^-2^. For stem weight, the maximum value was 683.92 g×m^-2^, and the minimum was 110.09 g×m^-2^. The coefficients of variation for the fresh weight of leaves and stems of *H. helix* were 33.44% and 34.51%, respectively. The contribution of the fresh biomass of herbaceous species to the total above-ground biomass was relatively low, ranging from 4 to 8%. The proportion of the dry above-ground biomass of leaves/stems of ivy and the dry biomass of forbs had a similar tendency.

**Table 3:** Functional traits of above-ground biomass of *H. helix* vegetative shoots.

Traits	Fresh above-ground biomass				Dry above-ground biomass				Fresh litter biomass	Dry litter biomass
	leaf	stem	forbs	total	leaf	stem	forbs	total		
mean, g×m^-2^	361.03	337.56	64.09	762.68	103.21	114.95	18.06	236.28	377.83	217.16
min, g×m^-2^	145.14	110.09	13.20	268.43	41.46	40.54	3.78	85.78	114.54	55.07
max, g×m^-2^	594.52	683.92	101.45	1379.89	235.60	243.92	28.98	508.50	1103.67	484.97
σ	120.37	116.49	28.76	286.91	41.04	48.52	7.09	104.01	227.66	93.59
V, %	33.44	34.51	44.89	37.62	27.52	32.56	39.26	44.01	60.25	43.09

### Water content of various plant parts and limiting environmental conditions

**Figure 1:**
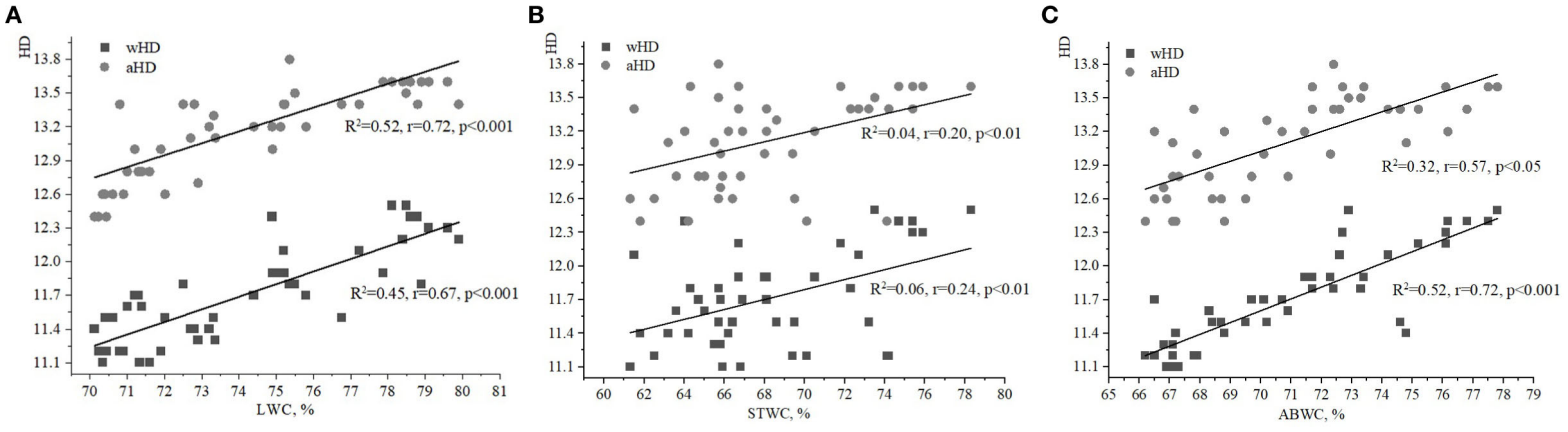
Relationships between water content of ivy parts and weighted/averaged means of soil moisture phytoindicator values (wHD/aHD): **(A)** – leaf water content (LWC), **(B)** – stem water content (STWC), **(C)** – above-ground biomass water content (ABWC).

**Figure 2:**
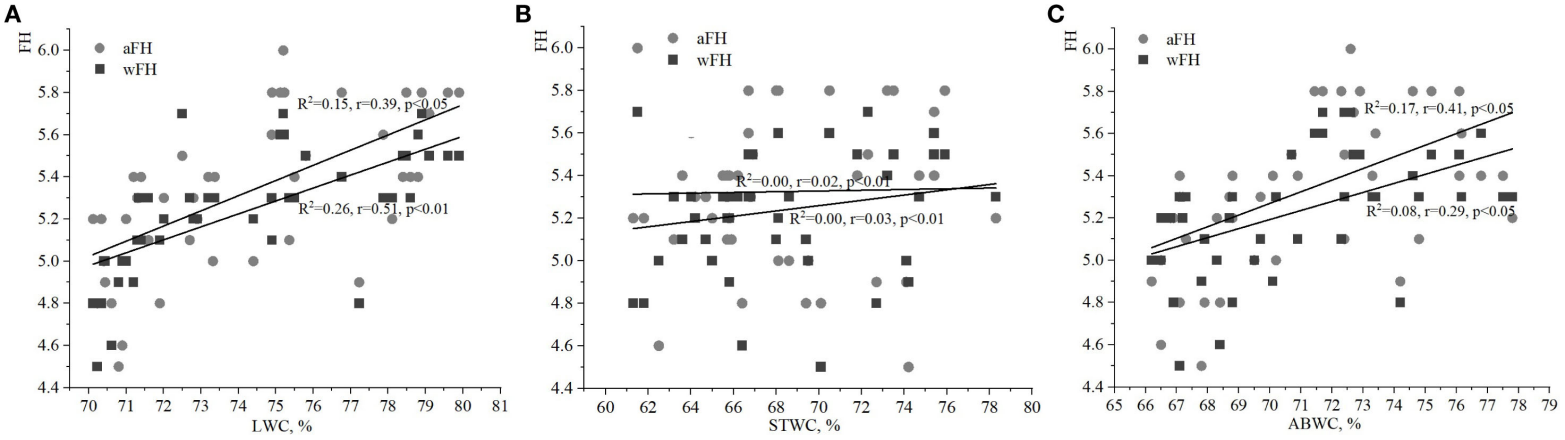
Relationships between water content of ivy parts and weighted/averaged means of variability of the soil moisture phytoindicator values (wFH/aFH): **(A)** – leaf water content (LWC), **(B)** – stem water content (STWC), **(C)** – above-ground biomass water content (ABWC).

**Figure 3:**
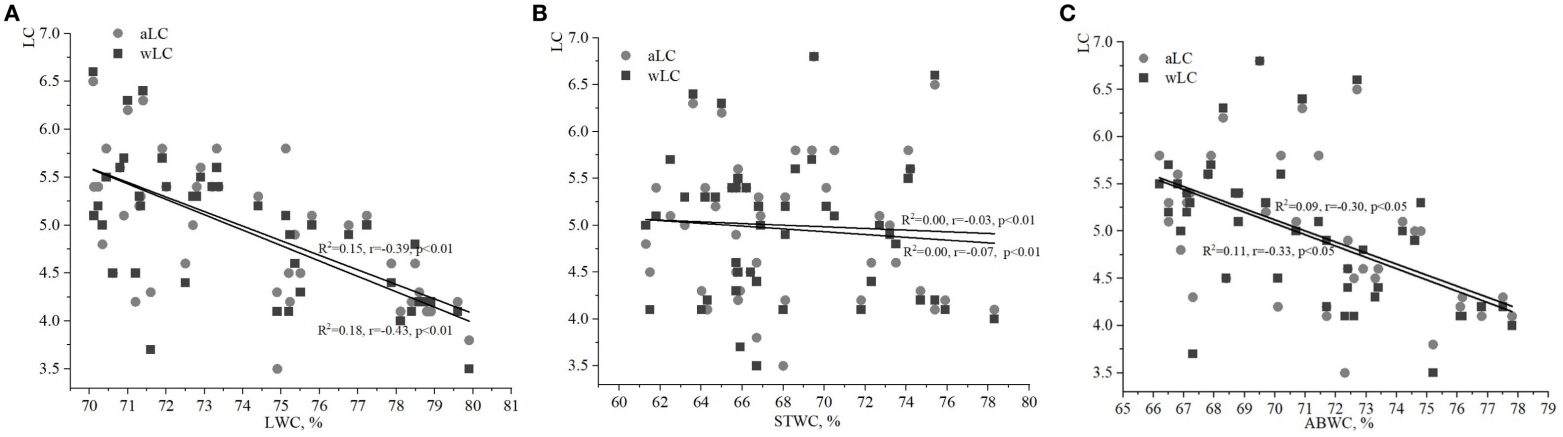
Relationships between water content of ivy parts and weighted/averaged means of soil light phytoindicator values (wLC/aLC): **(A)** – leaf water content (LWC), **(B)** – stem water content (STWC), **(C)** – above-ground biomass water content (ABWC).

The strongest relationship was found between the soil moisture index (HD) and the water content of various plant parts, particularly when compared to the indices of FH and LC. The LWC of ivy on the EPs of the Arboretum ranged from 70% to 80% (**Figures 1A**, **2A**, **3A**), with minimum and maximum values of 70.9% and 79.6%, respectively. These values ​​indicate a relative stability of LWC under the studied ecological conditions. Moderate correlations were observed between aHD and LWC (R^2^ **=** 0.52, r=0.72, p<0.001), as well as between wHD and LWC (R^2^ **=** 0.45, r=0.67, p<0.001).

The value of STWC ranged from 60 to 80%, indicating relative stability under the studied ecological conditions (**Figures 1B**, **2B**, **3B**). The extrema for STWC were 61.3% and 78.3%, respectively. However, no strong relationship was found between the soil moisture indicators (aHD and wHD) and STWC when compared to LWC. The similarity coefficients were 0.20 (p<0.01) for aHD and 0.24 (p<0.01) for wHD. The ABWC values ​​varied from 66 to 78% ​​(**Figures 1C**, **2C**, **3C**). The water content of the above-ground biomass of *H. helix* vegetative shoots demonstrated a linear relationship with the soil moisture phytoindicative value (**Figure 1C**). Additionally, the wHD (0.72, p<0.001) was found to be more sensitive to changes than the aHD (0.57, p<0.05). The data on LWC, STWC, and ABWC ​​suggest that the soil water availability for different parts of *H. helix* aligns with the field water capacity of the species during its initial growth stage (I stage).

The analysis of relationships between FH and LWC, STWS, and ABWC revealed weaker correlations compared to those observed with the soil moisture index (HD; **Figures 2A–C**). In particular, the relationships between LWC and aFH/wFH had correlations of 0.39 (p<0.05) and 0.51 (p<0.01), respectively (**Figure 2A**). Fixed correlations between ABWC and the variability of soil moisture were slightly lower than those found for LWC and aFH/wFH (**Figure 2C**). It should be noted that the correlation between wFH and ABWC was stronger (R^2^ = 0.17, r=0.41, p<0.05) compared to the correlation between aFH and ABWC (R^2^ = 0.08, r=0.29, p<0.05). Additionally, there was no significant correlation between the values of aFH/wFH and STWS (**Figure 2B**).

It is also important to analyze the correlation between the phytoindicative value of the limiting environmental factor, i.e. LC, and water content of various plant parts. The correlations between LC and both LWC and ABWC were weak (**Figures 3A, C**). However, the relationship between wLC and ABWC was stronger than between aLC and ABWC. No significant correlations were found between STWC and aLC/wLC in this study.

### Links between above-ground biomass of *H. helix* vegetative shoots and DLI and SLA

**Figure 4:**
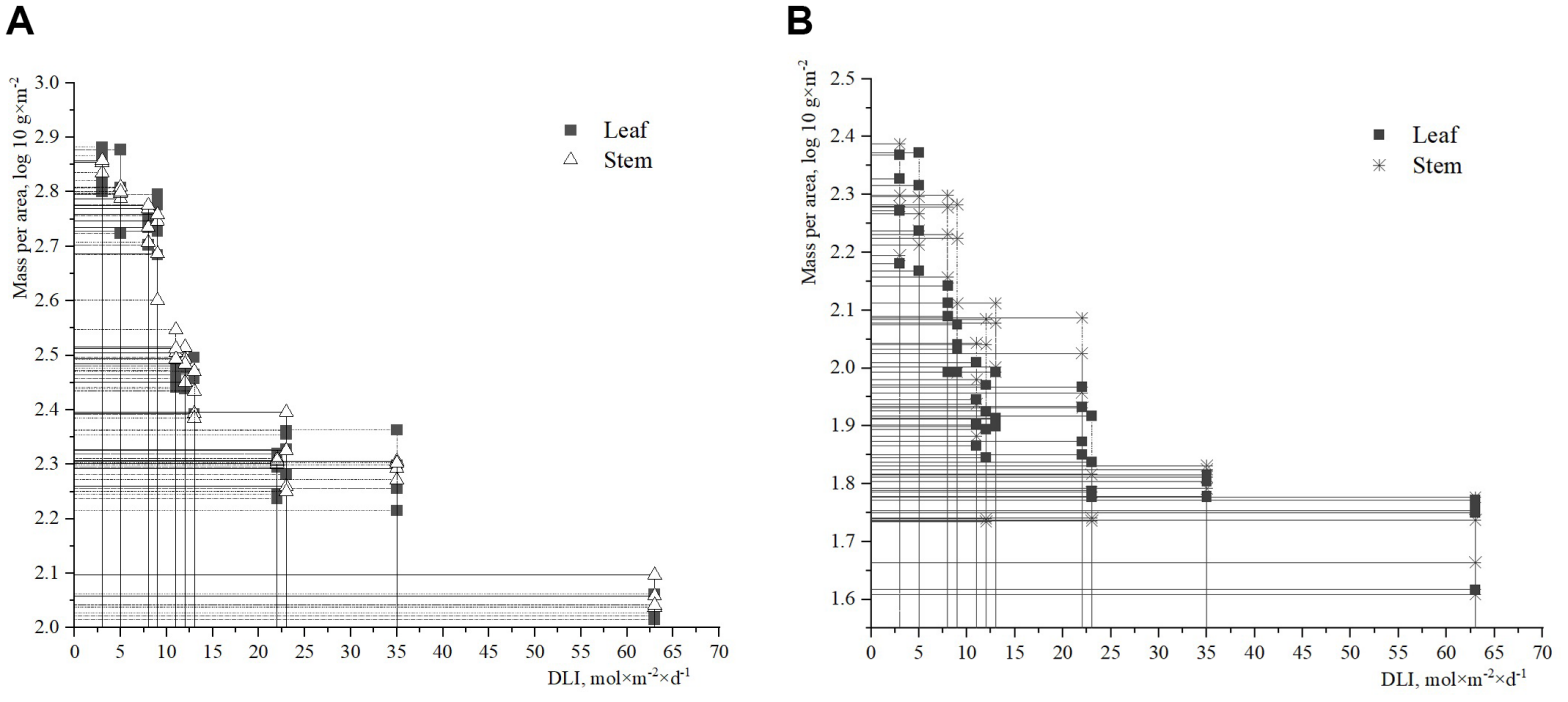
Above-ground biomass of *H. helix* (**(A)** – fresh; **(B)** – dry) per area and DLI.

The data collected revealed that the biomass of leaves and stems of *H. helix* per unit area peaked at a DLI value of 3–5 mol×m^-2^×d^-1^ (**Figure 4**). At this DLI range, the fresh leaf biomass per unit area was slightly higher than the fresh stem biomass. However, when the DLI exceeded 10 mol×m^-2^×d^-1^, the fresh stem biomass had higher values ​​than the fresh leaf biomass per unit area, compared to the lower DLI values of 3–5 mol×m^-2^×d^-1^. In addition, the lowest leaf and stem biomass was observed at a DLI value of 63 mol×m^-2^×d^-1^. A strong correlation between the fresh biomass of *H. helix* and DLI was identified (**Figure 4A**). It is worth noting that the correlation between leaf biomass and DLI was slightly stronger (R^2^ = 0.66, r=0.81, p<0.001) than the correlation between stem biomass and DLI (R^2^ = 0.58, r=0.76, p<0.001). The regressions of the dry weight of vegetative shoots against DLI (**Figure 4B**) also indicated significant relationships, i.e. leaf biomass (R^2^ = 0.58, r=0.76, p<0.001) and stem biomass (R^2^ = 0.48, r=0.69, p<0.001).

**Figure 5:**
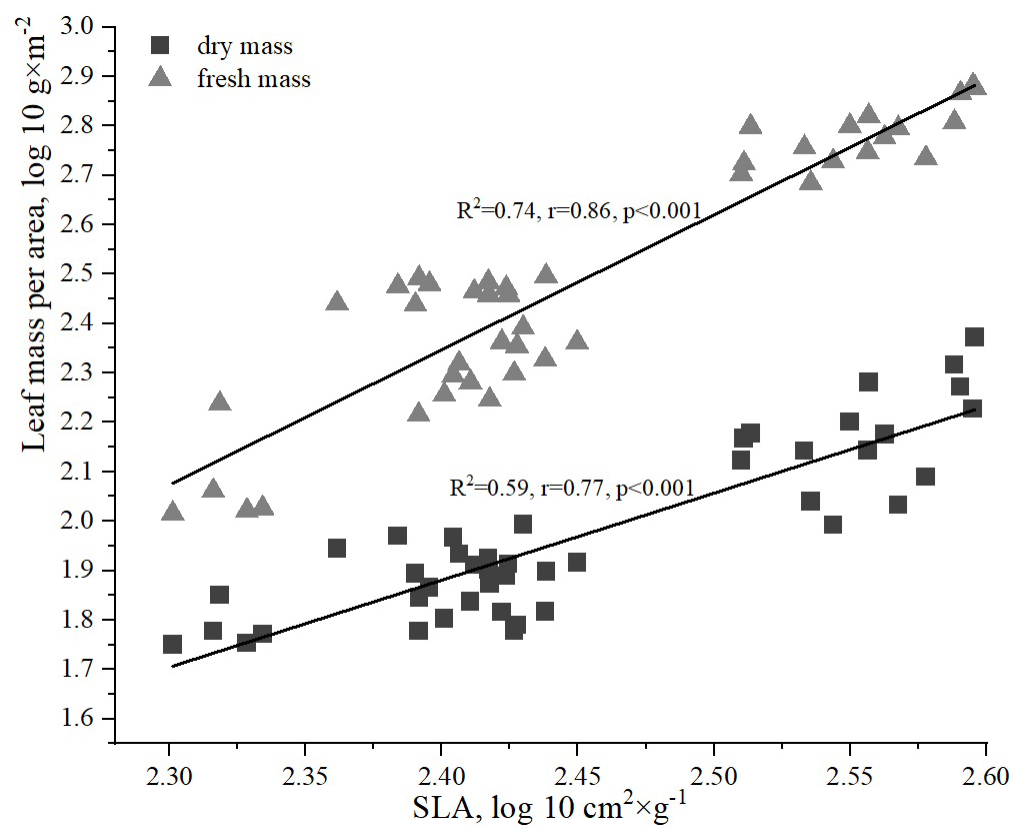
Relationships between dry and fresh leaf mass per area and specific leaf area (SLA).

The analysis of the relationship between the fresh and dry biomass of *H. helix* leaves revealed a strong correlation with the SLA (**Figure 5**). However, the relationship between the fresh biomass of ivy leaves and SLA was slightly stronger (R^2^ = 0.74, r=0.86, p<0.001) compared to the correlation between the dry biomass of ivy leaves and SLA (R^2^ = 0.59, r=0.77, p<0.001).

### Links between water content of *H. helix* vegetative shoots (LWC, STWC, ABWC) and DLI/SLA

**Figure 6:**
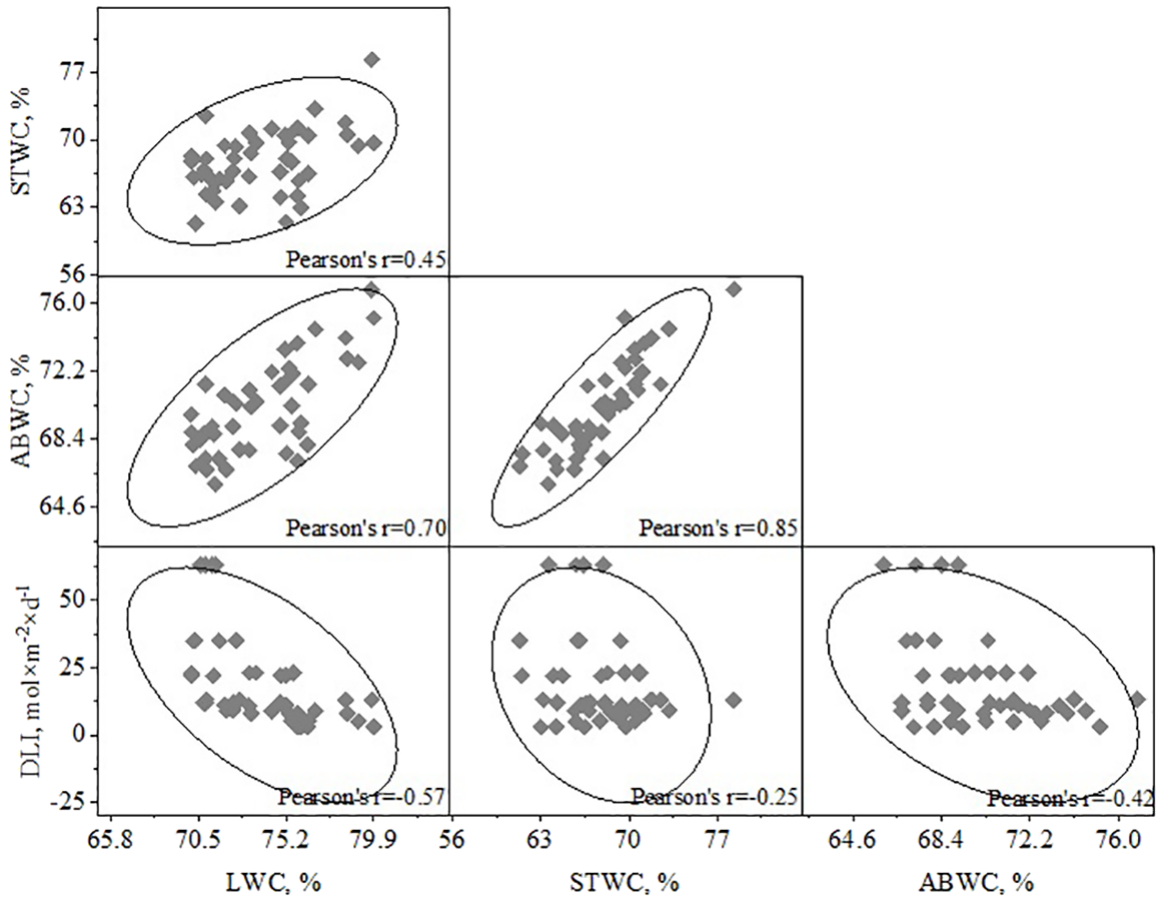
Dependence of water content of plant parts (LWC, STWC, ABWC) and DLI.

The analysis of the relationships between DLI and the water content of various parts of *H. helix* (LWC, STWS, ABWC) showed correlations of varying strengths (**Figure 6**). The strongest correlation was found between DLI and LWC (R^2^ **=** 0.32, r=-0.57, p<0.01). The correlation between DLI and ABWC weaker (R^2^ **=** 0.18, r=-0.42, p<0.05), while the relationship between DLI and STWC was even less strong (R^2^ **=** 0.06, r=-0.25, p<0.05). The correlations between STWC/ABWC and LWC/ABWC were quite as expected (R^2^ **=** 0.72, r=0.85, p<0.001 and R^2^ **=** 0.49, r=0.70, p<0.001, respectively).

**Figure 7:**
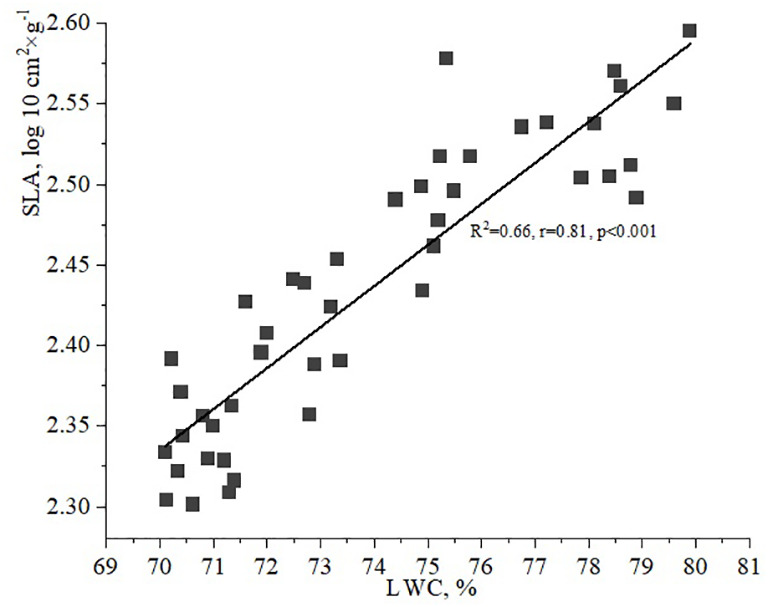
Relationship between leaf water content (LWC) and specific leaf area (SLA).

Additionally, it is important to examine the relationship between LWC and SLA. The data indicated that the SLA gradually increases as LWC values range from 70 to 80%. A strong correlation between LWC and SLA of vegetative leaves of *H. helix* was observed under the studied environmental conditions, with a correlation coefficient of 0.81 (**Figure 7**).

## Discussion

### Ecological implications and bioindicator potential

Disturbance of ecological balance can alter the relationship between biological diversity, ecosystem functions, and productivity (Isbell et al., 2018; Vančura et al., 2022). Patterns of biomass are influenced by phytodiversity, abiotic conditions, disturbance history, and/or forest type (Schnitzer and Bongers, 2011; Schnitzer et al., 2012; Viet et al., 2022). The demographic traits of dominant or abundant species are often more critical than general biological diversity indices in explaining biomass accumulation in the forest understory (Wasof et al., 2018).

*H. helix* is one of the most dominant lianas in Europe, where its increasing prevalence may significantly influence food webs and nutrient cycles in forest ecosystems in the future (Roeder and Meyer, 2022). In an environment changing due to climate change, where some species are becoming more dominant, litter composition will also vary. The changing litter composition in the forest alters ecosystem functioning (Badre et al., 1998). *H. helix* adds to the pool of fast decaying litter (relative to tree species) across seasons in the forest ecosystems (Roeder and Meyer, 2022). Interspecific competition for sunlight and plant nutrients may occur between *H. helix* and trees (Putz, 1991). The increasing dominance of *H. helix* populations will probably impact forest disturbances in the temperate zone of Europe as well. Previous studies of other authors have highlighted factors contributing to ivy’s success in European forests (Zotz et al., 2006; Paucã-Comãnescu et al., 2009). An increase in *H. helix* abundance has been observed both in forests and in urban ecosystems or orchards, indicating its adaptability to urban habitats. Based on the above, moderate impacts of ivy on tree population structure and diversity will likely result in transformations of forest composition and herb cover. Consequently, *H. helix* will also impact nutrient cycling both in forest ecosystems and urban parks (Matthews et al., 2016). Adult ivy individuals tend to thrive in forest areas with many ancient trees, where conditions are favorable for their growth. Nevertheless, completely overgrown (often shrubby) individuals are often noticed as well. There is evidence suggesting that the population density of ivy may be positively correlated with less-managed and close-to-natural forest areas in the temperate forests of Central Europe. However, this potential correlation should not be generalized to other habitats of *H. helix*, especially those of anthropogenic origin, such as urban parks, cemeteries, and gardens (Perring et al., 2020). In forest areas with high ivy abundance, a common silvicultural practice is to cut *H. helix* lianas at the base of the host tree (Roeder and Meyer, 2022).

Furthermore, the choice between direct or indirect methods of analyzing environmental conditions of the forest habitats, especially regarding the ecological dominance of the specific taxon, remains an ongoing discussion. Our results support Grime’s mass ratio hypothesis, which states that ecosystem stocks are primarily influenced by the relative abundance and plant functional traits of the most dominant species (Grime, 1998). Additionally, we confirmed the conclusions of Wasof et al. (2018) that only soil moisture indicators (HD; as mean phytoindicative values) showed a positive correlation with the above-ground biomass of ivy. This suggests that the dominance of *H. helix* is closely related to ecological changes. Overall, our results indicate that ivy serves as a sensitive bioindicator of soil moisture in the Kórnik Arboretum, and may be used as a biotic indicator of edaphic variables.

### Physiological responses to soil moisture

The relationship between soil moisture and the biomass of *H. helix* provides important insights into forest floor dynamics, particularly in temperate forests where moisture availability fluctuates due to both seasonal changes and long-term climate patterns. Our findings suggest that managing soil moisture levels can directly influence the growth and competitive dynamics of species like *H. helix*, which in turn affects overall forest health. Understanding the interactions between soil moisture, above-ground biomass water content, stem water content, leaf water content, and specific leaf area is important for explaining the water exchange in the soil–ivy–atmosphere system and biomass accumulation. Soil water content is an important element affecting plant physiology and morphology (Lower and Orians, 2003), regulating plant growth and metabolism (Huxman et al., 2004). The water content of various plant parts reflects species-specific adaptations to environmental changes across various biomes (Wang et al., 2022). In our study, we found closer relationships between LWC and edaphic traits compared to those of ABWC and STWC.

Indirect methods for estimating forest habitat features can be reliably applied in the cases of nitrogen and soil moisture (Halarewicz et al., 2021). We concur with these authors that direct and indirect methods should be used to assess environmental habitat specifics in relevant studies. Our results showed that only weighted means based on species cover are applicable for determining the relationships between the water content of various ivy organs and phytoindicative values of limiting environmental factors. A correlation between LWC and the soil moisture phytoindicative value (wHD) was established in this study.

LWC integrates the effects of atmospheric dryness, soil moisture, and plant drought tolerance, making it a critical indicator of plant productivity and health (Zhou et al., 2021). Variations in LWC is an adaptive response to instantaneous changes in limiting environmental conditions (Zhu et al., 2017; Zhang and Zhou, 2019). These studies proposed three response levels (slow-fast-slow) for LWC concerning soil moisture conditions, identifying a threshold value of 60.86 **±** 0.80%. This conclusion aligns with results based on leaf traits analysis (Yan et al., 2017), and partially supports the non-equivalence theory. Our data are consistent with the proposed response stages (Zhou et al., 2021) of LWC to soil moisture availability; a LWC value of 70–80% corresponds to Stage I of this response process.

High LWC values were related to greater temporal stability, demonstrating that lianas with more conservative economics are generally more stable over time. Results published by Krissakova et al. (2022) showed that *H. helix* belongs to the functional group characterized by high LWC values. Leaves with high LWC tend to be relatively tough (Smart et al., 2017) and are assumed to be more resistant to the pressures of abiotic factors than leaves with low LWC. The close relationship between SLA and LWC of vegetative *Hedera* leaves in the specified environmental conditions of the Arboretum was established in this study. However, we did not find evidence that increasing LWC correlated with a gradual decline in SLA. The observed LWC values ​​correspond to the field capacity of *H. helix* vegetative leaves and help to explain this relationship. We anticipate that a decline in SLA alongside an increase in LWC will become evident in Stages II and III of soil water availability as conditions approach the wilting point. Further studies of the dependence of water content of *H. helix* vegetative shoots and DLI values in different types of temperate forests ​​are extremely relevant. This area has received limited climatic attention and warrants future investigation to better understand how ecological changes affect the development and productivity of liana vegetative shoots.

Overall, LWC was weakly correlated with mean annual precipitation (Wang et al., 2022). As an important functional trait, LWC significantly impacts the leaf economics spectrum, with direct relationships to SLA (Garnier et al., 2001). Some authors have suggested that LWC may be a better sensitive indicator of forest above-ground biomass than SLA (Smart et al., 2017), especially since LWC is much easier to measure than SLA. These findings could enhance our understanding of vegetation development and provide insights into assessing the health of lianas under varying soil water conditions.

### Light availability and biomass allocation

Woody vines, such as *H. helix*, are among the plants that are most positively affected by increasing atmospheric CO_2_ concentrations. This enrichment significantly stimulates the vegetative growth of juvenile individuals in the understory, allowing them to establish more aggressively in low-light conditions. While mature plants may benefit less from elevated CO_2_ levels, the increased growth of younger individuals could enhance their ability to colonize the understory and potentially reach the forest canopy (Zotz et al., 2006). This ability may increase their impact on forest ecosystems, potentially leading to invasive behavior in the future. Research into the interaction between limiting abiotic factors, such as soil moisture and light, and the productivity of ivy is ongoing (Metcalfe, 2005).

Previous studies have highlighted the relationship between DLI and dry/fresh biomass in ivy (Yeh and Hsu, 2004; Huang et al., 2019). Correlation coefficients between DLI and ivy vegetative shoot biomass ranged from 0.66 to 0.96 (Yeh and Hsu, 2004). Notably, the relative stimulation of length and biomass increment of ivy vegetative individuals (600µl l^-1^) was indeed very pronounced in deep shade (+60%), about twice as much as in other forest strata (+30%) (Zotz et al., 2006). Our findings support these results, showing a 50% increase in above-ground biomass in shaded soil conditions compared to non-shaded areas. The curvilinear regression of shoot dry weight and soil light intensity observed in our study is consistent with previous research, which also highlighted genotypic variations of above-ground dry biomass response to amount of PAR in *H. helix* (Gislerod et al., 1989). A closer relationship between light availability of soil and *Hedera* phytomass was detected for environmental conditions of the Arboretum compared to artificial culture of ivy (Kim et al., 2012).

Another debatable issue is the leaf to stem biomass ratio for lianas under varying environmental conditions. Leaf mass per area (LMA) is a trait classically established as a sensitive predictor of plant functioning under environmental changes. Lianas contribute disproportionately more to forest canopy productivity and leaf area in relation to tree stems (van der Heijden et al., 2019), and allocate more above-ground biomass to leaves than to stems (Putz, 1983; Wyka et al., 2013; van der Heijden et al., 2019; van der Heijden et al., 2022). The conclusion of these authors regarding the disproportional ratio of leaf to stem biomass of *H. helix* only in conditions of intensive soil light was confirmed in this study.

Leaf traits, such as specific leaf area and leaf water content, are closely correlated to plant development and environmental conditions (Diaz et al., 2004). Variation in leaf morphological traits results from both environmental and evolutionary processes (Reich et al., 2003). Sunlight availability is a key environmental driver of the convergence of functional leaf traits in plant communities (Ackerly, 2003). The leaf functional traits that have the highest phenotypic plasticity could be more significant for leaf functioning in differing light (Rozendaal et al., 2006) and soil moisture conditions (Xu et al., 2020) than stem traits. Morphological traits of leaves reflect the leaf economic spectrum and adaptive response of plants to environmental conditions (Wright et al., 2004; Fang et al., 2017; Rodriguez-Perez et al., 2017).

The relationship between plant functional traits and subpopulation temporal stability (species biomass) related to plant traits (including SLA and LWC) was confirmed (Majekova et al., 2014). This stability is largely driven by the quantitative relationships between key functional traits, such as SLA and LWC. LWC is quantitatively correlated with SLA. Moreover, SLA will be quantitatively dependent on LWC, which can change as a function of growth phase. The increases in SLA attenuate with increasing LWC, demonstrating that leaf water potential sets a constraint on the maximum SLA that leaves can reach. The SLA was increased with LWC following Michaelis-Menten dynamics (Wang et al., 2022). Results of other authors suggested that the photosynthetic capacity of leaves decreased when LWC declined from 70 to 60% (Zhou et al., 2021). The backscatter coefficient had a direct correlation with LWC (R^2^ = 0.66) (Zhu et al., 2017). Increasing SLA values of *H. helix* leaves with decreasing DLI have been confirmed by the results of our and another study (Krissakova et al., 2022). SLA was positively related to soil moisture (R^2^ = 0.97) for shade leaves of *H. helix* (Doğan et al., 2015). Photoinhibition by high irradiance due to an increase in level of incident light were detected for *H. helix* individuals in deep shade (Oberhuber and Bauer, 1991).

SLA and LWC are key traits because they show the potential for plant development and reflect ecological adaptations in response to environmental transformation. Our results indicate that for slight ranges in soil moisture and soil light values, measurement of two indicators (LWC, SLA) is suggested for an awareness of the response and effect of trait relationships. Whether there exists a universal model that quantitatively forecasts all the pertinent links among leaf traits remains a debate issue.

## Conclusions

*Hedera helix* plays a key role in shaping the shrub layer and forest floor as a habitat-forming species. This study highlights the strong relationship between ivy productivity parameters and limiting environmental factors such as soil moisture and light availability, observed across clusters of ivy subpopulations by direct and indirect methods. Our findings reveal the important ecological role of *H. helix* as a key bioindicator of soil moisture and light in temperate forest ecosystems. Significant correlations were found between *H. helix*’s above-ground biomass and environmental factors, demonstrating the species strong adaptability to varying soil moisture and light levels. Notably, the disproportionate leaf-to-stem biomass ratio observed in shaded environments supports the hypothesis that *H. helix* prioritizes leaf production in low-light conditions to optimize photosynthetic efficiency. Furthermore, we established that water content in the leaves and stems of *H. helix* closely tracks soil moisture, underscoring the species capacity to maintain water balance under changing environmental conditions. These results deepen our understanding of liana dynamics in forest understories and provide practical knowledge for forest ecosystems management, particularly in regions where the expansion of *H. helix* may influence forest regeneration and structure.

## Data Availability

The original contributions presented in the study are included in the article/**Supplementary Material**. Further inquiries can be directed to the corresponding author.
