# Supplementary material for: Effects of limiting environmental conditions on functional traits of Hedera helix L. vegetative shoots
Source: Front Plant Sci. 2024 Nov 7;15:1464006. doi: 10.3389/fpls.2024.1464006 (PMC11578755; doi:10.3389/fpls.2024.1464006)
Supplement: Supplementary file 1 [file Table1.docx]

**Appendix 1**

**Allocation of experimental plots (1–11) on the territory of the Kórnik Arboretum, Poland** (figure was used in previous publication)


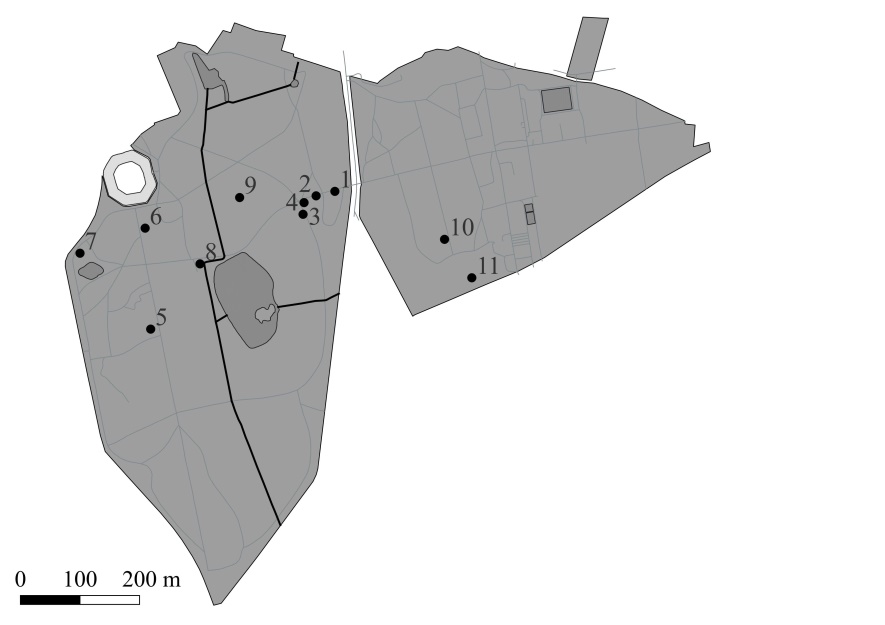


**Appendix 2**

**Characteristic features of the experimental plots**

| **No EP** | **Type and class of soil** | **Area of loci of *H. helix*, m^2^** | **Projective cover of**  ***H. helix*, %** | **Keystone species of herb layer** | **Keystone species of tree layer** |
| --- | --- | --- | --- | --- | --- |
| 1 | brown forest, sand | 1320 | 75 | *Aegopodium podagraria* L.  *Festuca rubra* L.  *Fragaria vesca* L.  *Malva sylvestris* L. | *Quercus robur* L.  *Fraxinus excelsior* L. |
| 2 | low moor peat, peal | 1674 | 95 | *Pteridium aquilinum* (L.) Kuhn *Mercurialis perennis* L.  *Geum urbanum* L.  *Aegopodium podagraria* L.  *Filipendula vulgaris* Moench. | *Quercus rubra* L.  *Gleditsia triacanthos* L. *Acer pseudoplatanus* L. |
| 3 | low moor peat, peal | 870 | 99 | *Pteridium aquilinum* (L.) Kuhn | *Taxus baccata* L.  *Picea abies* (L.) H. Karst. |
| 4 | low moor peat, peal | 1136 | 63 | *Melica uniflora* Retz.  *Stenactis annua* Nees  *Bromopsis ramosa* (Huds.) Holub  *Elytrigia repens* (L.) | *Tilia cordata* Mill.  *Acer platanoides* L. |
| 5 | anthropogenic soil, loamy sand | 1008 | 85 | *Mercurialis perennis* L.  *Fragaria vesca* L.  *Aegopodium podagraria* L. | *Taxus baccata* L.  *Picea abies* (L.) H. Karst.  *Pinus nigra* J.F. Arnold |
| 6 | anthropogenic soil, very fine sand | 876 | 88 | *Geum urbanum* L.  *Aegopodium podagraria* L.  *Bromus sterilis* (L.) Nevski  *Fragaria vesca* L.  *Urtica dioica* L. | *Carpinus betulus* L.  *Acer pseudoplatanus* L.  *Acer saccharinum* L.  *Larix decidua* Mill. |
| 7 | anthropogenic soil, sand | 1235 | 90 | *Melica uniflora* Retz.  *Glechoma hederacea* L.  *Impatiens parviflora* DC.  *Geranium robertianum* L.  *Humulus lupulus* L. | *Acer platanoides* L.  *Quercus robur* L. |
| 8 | anthropogenic soil, clay loam | 780 | 75 | *Bromus sterilis* (L.) Nevski  *Fragaria vesca* L.  *Impatiens parviflora* DC.  *Elytrigia repens* (L.)  *Ficaria verna* Huds. | *Fagus sylvatica* L.  *Acer platanoides* L.  *Betula pendula* Roth |
| 9 | gley-podzolic soil, sand | 845 | 70 | *Geum urbanum* L.  *Malva sylvestris* L.  *Galium verum* L.  *Urtica dioica* L.  *Galeobdolon luteum* Huds. | *Fraxinus excelsior* L.  *Quercus robur* L.  *Tilia americana* L. |
| 10 | erosional belt soil with accumulation process, very fine sand | 355 | 55 | *Bromus sterilis* (L.) Nevski  *Geum urbanum* L.  *Melica uniflora* Retz.  *Melica angustifolium* (L.)  *Elytrigia repens* (L.)  *Galium verum* L.  *Stenactis annua* Nees  *Urtica dioica* L. | *Taxus baccata* L.  *Carpinus betulus* L.  *Acer platanoides* L. |
| 11 | erosional belt soil with denudation process, silt | 1450 | 95 | *Geranium robertianum* L.  *Chamaenerion angustifolium* (L.) Scop.  *Galeobdolon luteum* Huds  *Impatiens parviflora* DC.  *Geum urbanum* L. | *Acer platanoides* L.  *Quercus rubra* L.  *Quercus robur* L.  *Betula pendula* Roth |
